# Assessing Spatial Accessibility of Community Hospitals for the Elderly in Beijing, China

**DOI:** 10.3390/ijerph20010890

**Published:** 2023-01-03

**Authors:** Jingya Luan, Yuhong Tian, Chi Yung Jim, Xu Liu, Mengxuan Yan, Lizhu Wu

**Affiliations:** 1 State Key Laboratory of Earth Surface Processes and Resource Ecology, School of Natural Resources, Faculty of Geographical Science, Beijing Normal University, Beijing 100875, China; 2 Department of Social Sciences, Education University of Hong Kong, Hong Kong, China; 3 China Academy of Urban Planning and Design (CAUPD), Beijing 100005, China

**Keywords:** spatial accessibility, aged population, spatial interaction model (SIM), community hospital, health equity, Beijing

## Abstract

Accessibility of health services signifies the quality and equitability of universal health provision. The hierarchical medical system recently implemented in China offers the policy instruments to improve medical services to the elderly in an aging society. As the critical primary care gateway, accessibility to community hospitals has significant impacts on people’s health. However, current research has paid little attention to spatial accessibility within walking distance of community hospitals, especially for the elderly. This study selected four districts with different urbanization levels in the rapidly developing Beijing metropolis. The spatial interaction model was applied to measure the accessibility of community hospitals for the elderly at the community level. An attractiveness index was computed based on key hospital traits. The results showed that: (1) community hospitals could cover 82.66% of elderly residents, and 77.63% of the communities were within walking distance. The served elderly proportion was relatively high in central urban areas and low in the suburbs. (2) The attractiveness indices of hospitals varied notably between districts, with higher values in more urbanized areas. (3) The spatial accessibility for the elderly of hospitals differed significantly between the four districts, with a descending gradient from central to suburban and rural areas, as indicated by the Gini coefficients and Lorenz curves. (4) The accessibility index was strongly related to the served elderly population and the hospital–residence distance. The findings provide policy directions to the government, including providing more primary-care resources to suburban and rural areas, building new community hospitals in identified provision gaps, upgrading some clinics to hospitals in rural areas, and planning hospitals according to the projected trend of the elderly population in terms of quantity and distribution. The considerable provision disparity between core urban, suburban and rural areas can be addressed by refined spatial health planning informed by research.

## 1. Introduction and Literature Review

Population aging refers to the increase in the proportion of the elderly relative to the total population. The phenomenon can be attributed to a decrease in young people and an increase in elderly people due to prolonged fertility decline and a rise in life expectancy [1]. Recently, the aging rate has accelerated worldwide, mainly due to widespread fertility and mortality decline, particularly in developing countries. The US Census Bureau’s 2016 publication, *An Ageing World: 2015*, reported that 8.5% (617 million) of the global population was above 65 in 2015 and was expected to double by 2050 [2]. In 2020, China’s population aged above 60 and 65 were 260 million and 190 million, accounting for 18.70% and 13.50% of the total population, respectively [3]. By 2050, the above-60 group will reach 480 million. Population aging has become an irreversible trend in China [4]. 

With an escalating aging trend, the elderly are anticipated to face inadequate medical-service problems due to the following: (1) the increasing urban elderly population will incur a high frequency of medical care, especially due to the common occurrence of chronic illnesses that demands recurrent medical attention; (2) due to the general decline in physical functions (especially slow and laborious locomotion due to impaired gait, balance and strength) and the associated mobility difficulties in using public transport vehicles, the elderly are more susceptible to the impact of distance between hospital and residence [5,6]. These inherent constraints have raised the need for more conveniently located medical facilities. 

Health care is a fundamental, indispensable and inalienable human right that allows people to live in dignity. Everyone has the right to equitable and accessible health care of a reasonable standard [7]. The elderly have compromised health status compared to younger people [8]. Most developing countries have considerable discrepancies in healthcare provision between urban and rural areas [9]. Unequal access to health care has been identified as a major cause of health inequality [10]. To improve the entire population’s health, the Chinese government issued the *Healthy China 2030* plan and the *Opinions on Promoting the Gradual Equalization of Basic Public Health Services*, aiming to establish equitable primary health services in all communities [11,12,13].

The Chinese government issued the *Guiding Opinions on Promoting the Construction of Hierarchical Medical System* in 2015 to alleviate the imbalanced distribution of medical resources. A hierarchical medical system includes a three-tier hospital framework. Primary hospitals, also known as community hospitals, directly offer public health and primary medical services to proximal community residents. The secondary hospitals provide a higher level of medical care to multiple communities. The tertiary hospitals offer the highest level of medical care and specialized clinical services to a large catchment and conduct medical education and research [14]. 

Most high-quality medical resources are concentrated in high-grade medical institutions in large cities, where residents near and far tend to seek medical treatment even for simple ailments. The undue centripetal effect causes overcrowding in such large hospitals to exacerbate further the elderly’s medical care difficulties [15]. The undesirable situation can be rectified by emphatic observation of the principle of initial diagnosis and screening in community hospitals and the two-way referral system. Thus residents’ medical treatment behavior can be rationalized and better organized, ultimately optimizing the use of precious medical resources. 

The elderly often suffer from chronic diseases requiring regular diagnosis and treatment. Community hospitals refer to local health service centers (stations), which can provide basic medical services (preventive care, regular diagnosis and treatments) to residents. Due to proximity to residents, community hospitals can play an important role in meeting a large proportion of the elderly’s medical care. Adequate provision of community hospitals can significantly reduce the risks associated with cardiovascular and other illnesses [16]. Critically, being located close to residents, they can preempt accessibility problems. They can fulfill crucial healthcare functions in more cost-effective and accessible ways to the elderly than high-grade hospitals [17]. During the COVID-19 pandemic, high-grade hospitals had a greater risk of infection among the elderly. The attractiveness of community hospitals to the elderly can be enhanced, for example, by improving services and shortening hospital–residence distance. Diverting more elderly patients to community hospitals can reduce the infection risk and alleviate overcrowding in high-grade hospitals. Improving the accessibility and capacity of community hospitals can also contribute importantly to establishing a holistic hierarchical medical system [18].

**Figure 1:**
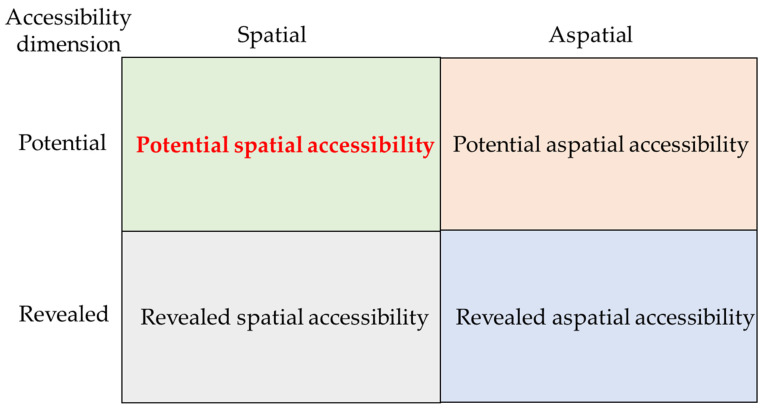
The conceptual framework for accessibility.

The accessibility concept can be traced to the classical location theory. It has always occupied a hot spot in geography, transportation, and urban planning studies. As early as 1959, Hansen [19] proposed the accessibility concept, defining it as the magnitude of the opportunities for interactions between nodes in a transportation network. Concerning medical accessibility, Khan [20] proposed a four-category scheme according to two dimensions, including potential spatial accessibility, potential aspatial accessibility, revealed spatial accessibility, and revealed aspatial accessibility (Figure 1). Revealed accessibility focuses on the actual use of medical services, while potential accessibility implies probable entry into health institutions but not necessarily using the provided services [20,21,22]. Spatial accessibility refers mainly to location, emphasizing the geographical barriers (distance or time) between providers (supply) and consumers (demand). Aspatial accessibility is associated with demographic variables and patient characteristics, including social class, income, age, gender and population health [23,24,25]. This study focused on potential spatial accessibility.

The literature has many studies analyzing hospital accessibility, which can be grouped under three aspects. Firstly, the accessibility of medical services is usually explored at the street and township levels or on an even smaller scale, such as individual buildings and population grid cells [26,27,28]. For example, Zheng et al. [29] analyzed the spatial accessibility of medical institutions in Kaifeng City (Henan Province, China) at the residential level. They provided a scientific basis for local governments to optimize the spatial structure of hospital resources. Based on a 1 km population grid unit, the accessibility of medical institutions in Beijing was investigated, generating results to enhance medical service planning [30]. 

Secondly, researchers have gradually realized the importance of nonspatial dimensions in assessing hospital accessibility [31]. They have explored the accessibility of health services to different sociodemographic groups. For example, Tang et al. [32] employed a two-step floating catchment area (2SFCA) statistical model to evaluate the elderly’s hospital accessibility in Taipei. Lee et al. [33] analyzed the accessibility to medical services and the underlying factors for persons with disabilities. They found that social and environmental factors had greater impacts than physical obstacles. Thus they suggested that the status of disabled persons should be improved through social policy instruments. 

Thirdly, hospital accessibility was studied at different levels of the medical institution hierarchy. For example, Huotari et al. [34] evaluated accessibility to tertiary hospitals in Finland using a gravity model based on population grid data and travel time estimates. Ruan and Zhang [35] constructed a potential model-based bus impedance traffic accessibility evaluation model and applied it to evaluate traffic accessibility to tertiary hospitals in Xi’an (Shaanxi Province, China). However, few studies have tackled the spatial accessibility of community hospitals for the elderly. 

The continued research on spatial accessibility in various application fields has generated a diverse range of new measurement methods. They include the shortest distance/time, the supply–demand ratio, the gravity model, and 2SFCA [9,17,36]. These methods only deal with the distance between facilities and consumers. They do not consider the optimal location of facilities and the attractiveness to consumers [30,37]. Therefore, it is difficult to evaluate the accessibility and attractiveness characteristics of community hospitals in different regions. The spatial interaction model can measure the optimal location of a service facility, with the proviso of consumers patronizing the nearest available facility. 

The space interaction model (SIM) was proposed by Huff [38]. It is analogous to Newton’s law of universal gravitation, which explains the aggregation behavior between two objects. The equilibrium force is related to spatial interactions [39]. The model considers the optimal facility location, the attractiveness of public services to residents, and the travel distance between the facility and residents [40,41,42]. Compared with others, this model is relatively simple yet versatile. Suitable for the small-scale study of spatial accessibility, the SIM has been widely applied to universities, playgrounds, parks, etc. [43,44,45]. Since this study concentrates on the accessibility measure of community hospitals within walking distance of the elderly, it belongs to small-scale research. As the model can consider multiple indicators in calculating facility attractiveness, it can comprehensively reflect community hospitals’ capabilities. Therefore, this study adopted the SIM to evaluate the spatial accessibility of community hospitals in different districts of Beijing to better serve the elderly within walking distance.

As China’s capital, Beijing has experienced fast economic development. Its population has lapsed into a relatively advanced aging stage. The city’s 2020 census recorded 4.299 million people aged >60, at 19.6%, exceeding the 18.7% national level [3,46]. This record is close to the lower limit of “20%” of the moderate aging category proposed by the United Nations [47]. The city’s aging situation is anticipated to become more acute in due course. The accelerated aging rate will raise the health-service demand of the elderly to surpass the supply and challenge Beijing’s socioeconomic development. In addition, as the pilot city for the hierarchical diagnosis and treatment program, Beijing had over 2200 community hospitals by late 2020 [48]. This infrastructural development has furnished key health services to the elderly. However, previous studies have often focused on the accessibility to high-grade hospitals rather than community hospitals [49]. The accessibility to community hospitals within walking distance for the elderly in different areas remains unclear. Given this knowledge gap, this study proposes the following research questions: (1) Can the current planning and location of community hospitals meet Beijing’s goal of providing community hospitals within walking distance to the elderly in different areas? (2) Are there regional differences in the attractiveness and accessibility of community hospitals for the elderly living within walking distance? (3) Are there inequities in access to community medical resources in regions with different urbanization levels?

To solve the above research problems, this study selected four functional areas with different urbanization levels in Beijing. The SIM explored the spatial accessibility of community hospitals and associated factors for the elderly. We then analyzed the attractiveness of community hospitals and identified areas with a relative shortage of primary medical services. This study aims to improve the spatial accessibility of community hospitals from the perspective of the elderly. The solutions could allow rational allocation and utilization of limited medical resources and promote overall population health. The findings have theoretical and practical applications for alleviating the healthcare problems caused by aging, meeting the medical needs of the elderly, fostering hierarchical diagnosis and treatment, and achieving a more equitable provision of basic public service.

## 2. Study Data and Methods

### 2.1. Study Area

**Figure 2:**
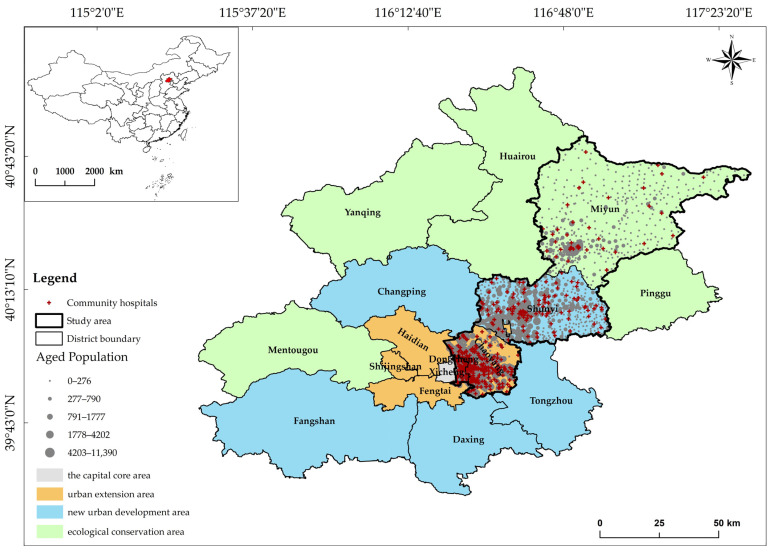
Distribution of community hospitals and the aged population in the study areas in Beijing.

Beijing is located at 115.7–117.4° E and 39.4–41.6° N. The capital city is China’s political, cultural, international exchange, and science and technology innovation center. It has 16 districts and a total area of 16,410.54 km^2^. According to the urbanization level, the metropolis is divided into four functional areas (Figure 2): the capital core area, urban extension area, new urban development area, and ecological conservation area [50]. The *Special Plan for Medical and Health Facilities in Beijing (2020–2035)* evaluated the distribution of medical facilities. The uneven regional distribution is evident. The high-grade medical and health resources are mainly concentrated in the central urban area, accommodating over 80% of tertiary hospitals. Shunyi, Daxing and other new urban development areas, and Miyun and other ecological conservation areas, have insufficient medical and health resources [51]. 

By the end of 2020, the total permanent population of Beijing was 21.89 million, of which 4.299 million were aged ≥60, with an aging rate of 19.6% [46]. Four administrative districts of Dongcheng and Chaoyang (city center with high urbanization level), and Shunyi and Miyun (suburbs with low urbanization level), representing Beijing’s four functional areas, were selected as study areas (Figure 1). The districts have different aging rates, with Dongcheng, Chaoyang and Miyun exceeding 20% and Shunyi at 16.5% (Table 1). Thus, the elderly population is unevenly distributed among districts, with a concentration in central and suburban urban areas. 

### 2.2. Data Collection and Processing

#### 2.2.1. Data on Community Hospitals and Administrative Boundaries

The study areas have 410 community hospitals, including 55 in Dongcheng, 212 in Chaoyang, 104 in Shunyi and 39 in Miyun. The locations of the community hospitals were extracted from the AutoNavi map in 2020 (https://lbs.amap.com/ accessed on 10 June 2022). The key traits of community hospitals, including construction areas, number of medical staff, number of departments, and number of family doctor teams, were derived from the government’s district websites (https://www.cmacsubaru.com/so/index.html accessed on 10 June 2022), the District Health Commission Report (http://wjw.beijing.gov.cn/), 99 Health Network (https://yyk.99.com.cn/ accessed on 10 June 2022), and WeChat public accounts. Field investigations supplemented and validated the data. The provincial, county, street and village boundaries were derived from the Resource and Environment Science and Data Center of the Chinese Academy of Science (https://www.resdc.cn/ accessed on 10 June 2022).

#### 2.2.2. Population Data at the Community/Village Level

The population data included the number of elderly people in communities/villages within walking distance of community hospitals. The residential community data in Dongcheng and Chaoyang were derived from Lianjia.com (https://sn.lianjia.com/ accessed on 10 June 2022). By writing a Python program, the 2020 data of all residential communities in the two districts were obtained, including the community name, location, area, number of households and coordinates (Baidu map coordinate system). A total of 2984 residential communities were obtained, comprising 2,018,247 households. Since the permanent population of the two districts was 4,161,289 people, each household had about 2.062 persons. The community’s elderly population was estimated based on the total number of households, according to the proportion of the elderly population in the Dongcheng and Chaoyang districts (Table 1). 

Shunyi and Miyun are located in the suburbs, where the administrative units are mainly villages. We downloaded the 100 m resolution population raster data from the Wordpop website and projected it with the geographic coordinate system WGS1984. The data unit is the number of people per pixel. We summarized the raster values with village boundaries. First, the raster values were corrected with the permanent population data of streets/towns in the statistical yearbook. We then summarized by village boundaries to obtain the population of 919 villages in Shunyi and Miyun, replacing the population center of gravity with the center of mass of administrative villages. The preprocessed data were tested with the known village population data obtained in the actual survey process, yielding high reliability of the tested data. 

### 2.3. Defining the Scope of Community Hospital Services

**Figure 3:**
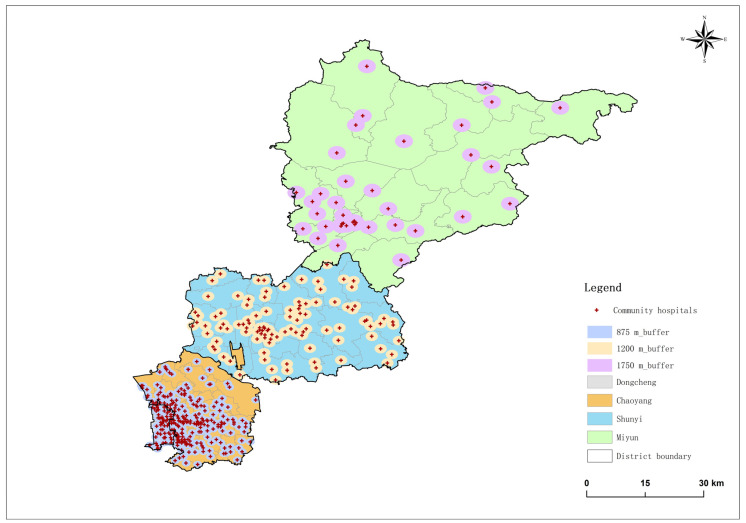
Walking distances to community hospitals in four districts.

The walking speed of the elderly is usually documented as 3.5 km/h [52,53]. According to the latest planning and setting of community health service centers and stations proposed by Beijing, community health services must be within a 15 min walk for residents in urban areas, 20 min for residents in suburban plains, and 30 min for residents in mountainous areas [54]. Accordingly, the service radius of community hospitals in Dongcheng and Chaoyang (urban) for the elderly was defined as 875 m, Shunyi (suburban plain) as 1200 m, and Miyun (suburban mountain) as 1750 m. Figure 3 shows the distribution of different walking catchments of community hospitals. 

### 2.4. Model for Spatial Accessibility Measurement of Community Hospitals

#### 2.4.1. Determining the Accessibility Measures

Inspired by Newton’s law of universal gravitation, the spatial interaction model [55] can be expressed by Equation (1):

(1) $$A_{ij}\;=K\times \left(R_{i}\times P_{j}\right)÷d_{ij}^{\propto }$$

where $A_{ij}$ represents the interaction between *i* and j; *K* is a general constant; $R_{i}$ is the supply strength of $i;\;P_{j}$ is the demand strength of $j;\;d_{ij}$ is the distance between i and j; ∝ is the distance parameter, ∝=2 [43,56]. $A_{ij}$ is a cumulative value that is directly proportional to the strength of $R_{i}\;\mathrm{and}\;P_{j}$ and inversely proportional to the distance between i and j. 

To analyze the interaction between a community hospital and the population it serves, the model could be modified to Equation (2). $A_{i}$ as maximum indicates better accessibility of a community hospital to residents.

(2) $$A_{i}=K_{i}\times \underset{j=1}{\sum^{n}}P_{j}÷d_{ij}^{2}$$

where $A_{i}$ represents the spatial accessibility of community hospitals; $K_{i}$ is the general constant; $P_{j}$ is the number of elderly people in community j within the scope of community hospital i; n is the number of communities/villages within walking distance of community hospitals; $d_{ij}$ is the Euclidean distance between community hospitals and the community.

#### 2.4.2. Defining the Community Hospital Attractiveness Index

$K_{i}$ represents the main characteristics of community hospitals that attract the elderly, labeled as the attractiveness index or supply capacity. It is generally expressed by a basket of key indicators, such as the grade of medical institutions, building area, number of beds, number of medical staff, number of family doctor teams, and number of departments [57,58,59]. However, during the actual investigation, due to the impact of the COVID-19 pandemic, most community hospitals did not accept hospitalization. Thus, the number of beds was removed in this study. *K* is expressed by Equation (3):

(3) $$K_{i}=[(W_{1}*R_{Ai}+W_{2}*R_{Pi}+W_{3}*R_{Di}+W_{4}*R_{FDi})÷4]*100$$

$\mathrm{where}\;K_{i}$ is the attractiveness index or supply capacity of community hospitals i; $R_{Ai}$ is the relative value of the building area of the community hospital i; $R_{Pi}$ is the relative value of the number of medical staff; $R_{Di}$ is the relative value of the number of departments; $R_{FDi}$ is the relative value of the number of family doctor teams. 

A former study indicated that the number of medical staff, departments and building area were weighted as 0.1003, 0.0679 and 0.0465, respectively, in defining the supply capacity of hospitals [60]. During the field surveys, we communicated with elderly persons and found that the importance of the number of family doctor teams for the elderly was higher than the building area index, but the importance of the number of departments was not as high. So, this index’s weight was calculated by averaging the weights of the number of departments and building area. Thus, the four factors were weighted as follows: number of the medical staff at 0.1003, number of departments at 0.0679, number of family doctor teams at 0.0572, and hospital building area at 0.0465. 

We divided the *K* values of the four districts into five grades according to the natural break categories method to analyze the attractiveness level of the community hospital. The higher the grade, the stronger the attractiveness. 

### 2.5. Lorenz Curve and Gini Coefficient

The Lorenz curve, which reflects the fairness of the distribution of income or wealth in society, is widely used to study fairness in allocating medical resources [61,62]. It is drawn with the cumulative percentage of the population or geographical area on the *x*-axis and the corresponding cumulative percentage of resources on the *y*-axis. The diagonal denotes a perfectly equal line. Absolute fairness is achieved when the Lorenz curve coincides with the perfect-equality line. The greater the curvature of the curve, the less equitable the resource distribution, and vice versa.

The Gini coefficient is a quantitative index based on the Lorenz curve, which can evaluate the fairness of a community’s medical resource allocation [18]. It can be calculated by Equation (4):

(4) $$G=1-\overset{n}{\underset{i=1}{\sum }}\left(X_{i}-X_{i-1}\right)\left(Y_{i}+Y_{i-1}\right)$$

where G represents the Gini coefficient; $X_{i}$ represents the cumulative percentage of the population; $Y_{i}$ represents the cumulative percentage of the corresponding medical resources; n is the total number of districts and counties, i = 1, 2, 3 …, n. At present, there is no specific evaluation standard for the Gini coefficient in the health field. Thus, economic standards are used as evaluation criteria. The value of the Gini coefficient lies between 0 and 1; at 0, the distribution of medical resources is absolutely fair; at 1, it is completely unequal. *G* < 0.2 indicates that the medical resource configuration is optimal, *G* = 0.3 to 0.4 is normal, 0.6 > *G* > 0.4 is an alarming state, and *G* > 0.6 is a highly unfair and dangerous state. The larger the Gini coefficient, the more unfair the allocation of medical resources. This study used spatial accessibility values, established in light of walking distance of community hospitals in each district, to calculate the Gini coefficient. 

## 3. Results

### 3.1. Scope of Community Hospital Services

As shown in Table 2, Beijing’s community hospitals can serve 77.63% of the community and 82.66% of the elderly within walking distance. Overall, most community hospitals are accessible to elderly residents. However, there are significant regional differences. The community hospitals in Dongcheng, situated in the city center, can cover 96.14% of the community and 98.23% of the elderly. Of the 1121 communities within walking distance in Dongcheng, 964 (86%) have walking-distance access to two or more community hospitals. For example, the elderly in the Dingshengyuan community can walk to the Jingtaixili Community Hospital, Wangtan Community Hospital, and Fulaiyin Community Hospital. The elderly at 28 Minzhu East Street can choose four community hospitals within walking distance. The community hospitals in Chaoyang, situated in the city center, can cover 86.47% of the community and 89.7% of the elderly. Of the 1572 communities within walking distance in Chaoyang, 1146 (73%) can walk to two or more community hospitals. For example, the elderly in the Shuiduizi Xili community can walk to eight community hospitals. 

However, the community hospitals in Shunyi, situated in the suburbs, cover only 40% of the community and just over half of the elderly. Of the 180 communities within walking distance of community hospitals, only 44 (24%) can walk to two or more hospitals, mainly located in Shunyi urban areas. Community hospitals in suburban Miyun can serve 33.55% of the community and 67.15% of the elderly group. Of the 157 communities within walking distance, only 71 (45%) can walk to two or more community hospitals. These communities are generally located at the intersection of two or more hospital catchment areas. They are usually considered transitional areas for the supply of primary medical resources. Many elderly villagers cannot reach community hospitals within walking distance and have difficulties seeking medical attention. 

### 3.2. Attractiveness of Community Hospitals

**Figure 4:**
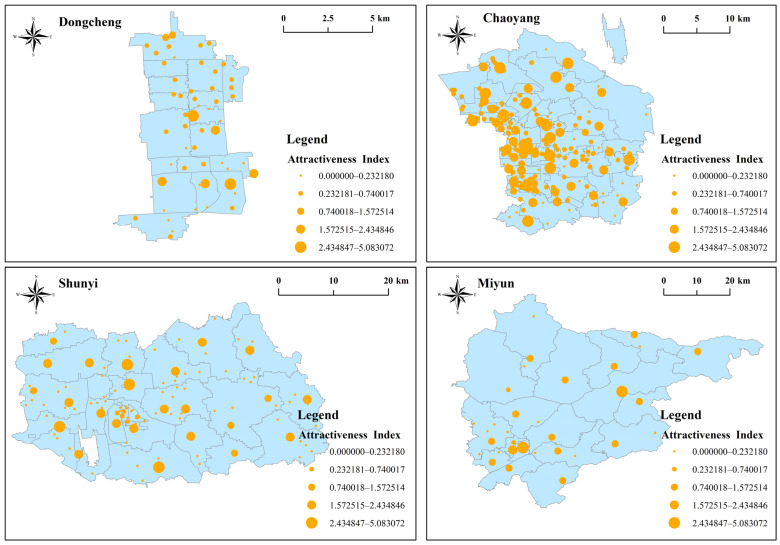
Attractiveness index of community hospitals within walking distance of four districts.

Figure 4 shows the attractiveness index of community hospitals in four districts. The attractiveness index was classified into five levels (Figure 3): very high, high, medium, low, and very low. The average attractiveness index was 0.68, with only 107 community hospitals (circa 26.1%) higher than the average. Therefore, the attractiveness of community hospitals in Beijing was low overall. 

The four districts demonstrated varied results. Eighteen (37.72%) community hospitals in Dongcheng had very low attractiveness indices. The Tieguluba ($K_{i}$ = 0.17), Xiaopaifang Hutong ($K_{i}$ = 0.17) and Fahuasi Community Hospitals ($K_{i}$ = 0.17) had the lowest indices. Two (3.64%) venues, Chaoyangmen ($K_{i}$ = 3.18) and Longtan Community Hospitals ($K_{i}$ = 3.795), had very high attractiveness indices. 

As many as 54 (25.47%) community hospitals in Chaoyang had very low attractiveness indices, contrasted with 15 (7.08%) at a very high level. Balizhuang Second ($K_{i}$ = 4.57), Sun Palace ($K_{i}$ = 4.39), Xiaohongmen ($K_{i}$ = 3.07) and Wangjing Community Hospitals ($K_{i}$ = 3.07) had very high attractiveness indices. 

A total of 76 (73.08%) community hospitals in Shunyi had very low attractiveness indices; Zhuangziying Community Hospital had the lowest ($K_{i}$ = 0). On the other hand, three (2.88%) community hospitals had a very high attractiveness index, namely Niulanshan Town ($K_{i}$ = 2.92), Mapo Town ($K_{i}$ = 2.74) and Houshayu Town Community Hospitals ($K_{i}$ = 5.08). 

Miyun had 19 (48.72%) community hospitals with very low attractiveness indices. However, it had two (5.12%) at a very high level, namely Gulou ($K_{i}$ = 3.09) and Taishitun Town Community Hospitals ($K_{i}$ = 3.03). In summary, the attractiveness indices of most community hospitals in the study areas were comparatively low, and the overall attractiveness to elderly clients was inadequate. The attractiveness of community hospitals in urban areas was relatively good, but it was poor in most suburban rural areas.

### 3.3. Accessibility Analysis of Community Hospitals

**Figure 5:**
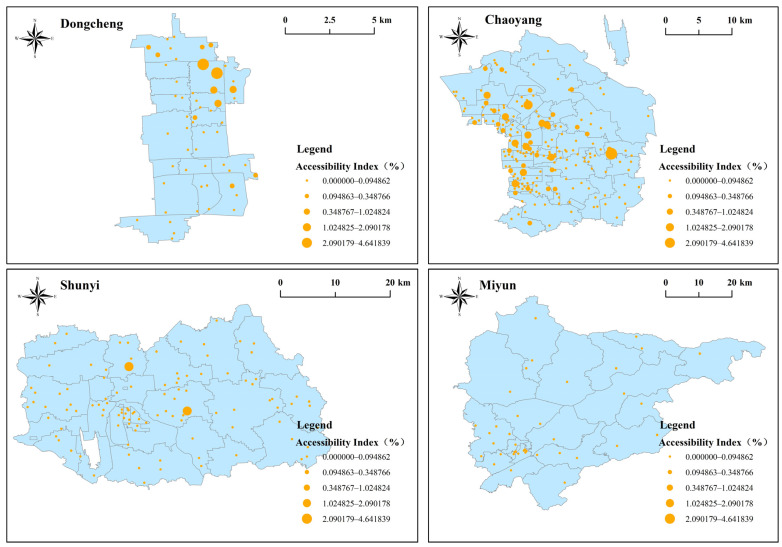
Accessibility index of community hospitals within walking distance of four districts.

The accessibility analysis results were divided into five categories: very high, high, medium, low, and very low. Figure 5 shows that a notable proportion of community hospitals had very low accessibility. Table 3 indicates the average accessibility indices at 0.0857% for all study areas, with Dongcheng at 0.2439%, Chaoyang at 0.0880%, Shunyi at 0.0276%, and Miyun at 0.0054%. The accessibility to community hospitals in central urban areas was significantly higher than in the suburbs. Thus, the elderly living in the central areas had more convenient access to community hospitals.

In Dongcheng, the Qinglong ($A_{i}$ = 4.6418%) and Min’an ($A_{i}$ = 4.5436%) Community Hospitals had the highest accessibility indices. The Dongheyan ($A_{i}$ = 0.0047%) and Longtan Beili ($A_{i}$ = 0.0044%) Community Hospitals had the lowest accessibility indices. In Chaoyang, the Changying ($A_{i}$ = 2.0902%), Wangjing ($A_{i}$ = 1.3644%) and Datun ($A_{i}$ = 1.0248%) Community Hospitals had the highest accessibility indices. The Jinzhan Second ($A_{i}$ = 0), Heizhuanghu ($A_{i}$ = 0) and Shibalidian Hengjiezi ($A_{i}$ = 0) Community Hospitals, far from the central urban area, had the lowest accessibility indices. In Shunyi, the Niulanshan Town ($A_{i}$ = 1.2527%) and Nancai Town ($A_{i}$ = 1.2920%) Community Hospitals had the highest accessibility indices. The Zhuangziying Community Hospital had the lowest accessibility index ($A_{i}$ = 0). In Miyun, the Guoyuan Community Hospital ($A_{i}$ = 0.0914%) had the highest accessibility index. Overall, the accessibility indices were low. It was relatively convenient for the elderly in some central urban areas and a few suburbs to seek medical treatment to achieve the basic goals of community hospitals. Nevertheless, most elderly living in the more rural parts of the suburbs could not reach community hospitals within walking distance. 

### 3.4. Measuring Inequality with the Gini Coefficient and the Lorenz Curve

**Figure 6:**
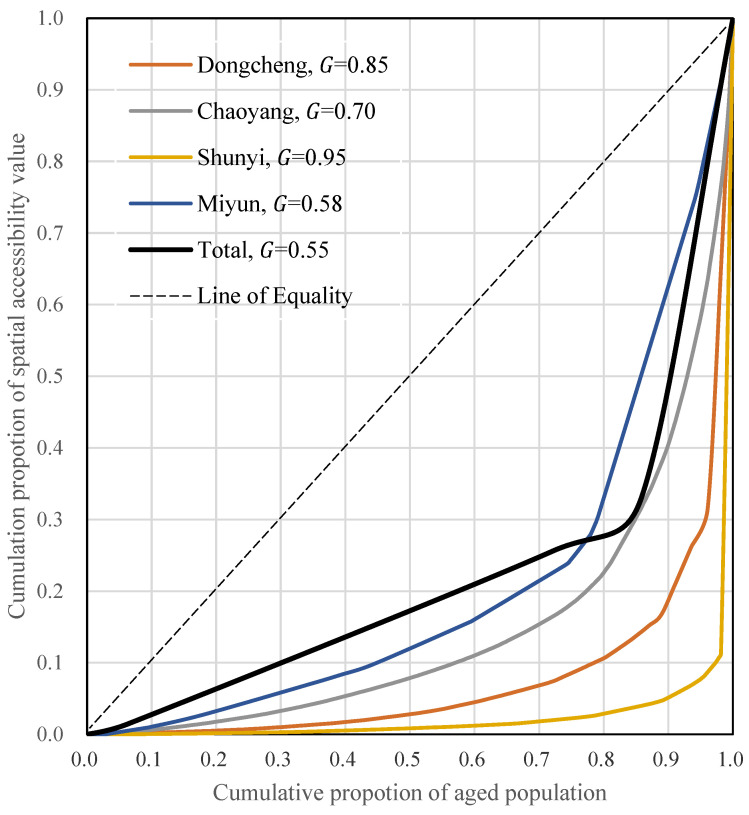
Lorenz curves and Gini coefficients for spatial accessibility of community hospitals by the aged population in four districts.

Figure 6 shows the Lorenz curves and Gini coefficients of spatial accessibilities for community hospitals in different districts. The overall Gini coefficient for accessibility to community hospitals was high, at 0.55, with significant differences among districts. Accessibility to community hospitals also varied considerably within the districts. The largest intra-district discrepancies occurred in Shunyi, followed by Dongcheng, Chaoyang and Miyun, with Gini coefficients of 0.95, 0.85, 0.70 and 0.58, respectively. The smallest intra-district differences in Miyun were mainly due to overall low accessibility. The equitable distribution model calls for government reallocation of community medical resources to provide more equitable community hospital accessibility to the elderly. 

## 4. Discussion and Conclusions

### 4.1. Advantages and Limitations of the Spatial Interaction Model

The spatial interaction model (SIM) is often used to assess the optimal location of a facility, the attractiveness of public services to residents, and the travel distance between a facility and residents. In this model, researchers can flexibly adjust the parameters K and∝ according to research purposes. The model has been widely applied to assess universities, playgrounds, shops, markets, parks, etc. [43,44,45,63]. 

This study used the weighted average of multiple indicators to compute the compound attractiveness index K. Similar studies mainly used simple indicators, such as the number of medical staff and beds [37,64]. The SIM enjoys the advantages of encompassing more indicators, improving the attractiveness evaluation of medical resources, and differentiating attractiveness among regions. The results are comparatively more objective. However, our model only considered the location and interaction between community hospitals and client groups. The accessibility of community hospitals in suburbs or villages might be affected by other factors, such as the dispersion of communities and poor traffic conditions. Future studies can incorporate these additional parameters into the model. 

### 4.2. Scope and Attractiveness of Community Hospital Services

This study indicated that the community hospitals in the central urban areas could serve more communities and a larger proportion of the elderly population. The elderly in most communities could walk to two or more community hospitals. In contrast, suburban community hospitals could only serve just over half of the elderly population within walking distance, and few elderly people in suburban towns could walk to two or more community hospitals. These findings were consistent with previous studies that central urban areas have easier access to medical resources than suburbs [65].

Moreover, community hospitals were unevenly distributed in the suburbs, where some elderly people could not walk to reach them. The deprived population segment had to use other transportation means to access medical services, creating difficulties for the elderly with mobility impairments, disabilities and poor physical health. In most villages, primary medical services for the elderly were poorly accessible, which may threaten their health. In Beijing, 214,436 (17.34%) elderly citizens, largely dwelling in suburban and rural areas, could not access community medical services on foot. This situation is similar to the large Shenzhen metropolis in south China, where the uneven distribution of medical services in some parts has deprived over 30% of residents easy access [66].

This study also showed that the attractiveness of Beijing’s community hospitals was unsatisfactory, regardless of central urban or suburban location. This result is consistent with previous findings [67,68]. In China, community hospital developments depend largely on the government’s financial subsidies and competition from surrounding medical institutions. Due to urban planning policies, the suburban government receives extra financial subsidies from the Beijing government. Currently, the few high-level hospitals in the suburbs have limited competition, so their attractiveness index was comparable to that in the central urban area. However, the difference in the attractiveness index between community hospitals in the central urban area was smaller than in the suburbs, mainly caused by the limited supply of suburban community hospitals. 

In recent years, the national government has greatly improved primary medical care. For example, the hierarchical medical system and equalization of basic public services in urban and rural areas have been implemented. However, China’s primary medical and health service system is still relatively weak compared to developed countries. The main concerns in the health infrastructure include the uneven professional and technical capabilities between regions and the limited attractiveness of community hospitals to residents. Suitable measures could be introduced to upgrade the healthcare needs of prevention, treatment and rehabilitation in suburban and rural areas, especially for the elderly. 

### 4.3. Spatial Accessibility Factors of Community Hospitals

Allocation of healthcare resources has to consider multiple factors, such as accessibility and equity. Spatial accessibility often plays an important role. The surrounding residents welcome community hospitals with high accessibility. This study showed that the overall accessibility of community hospitals in the study area was low, with only a few communities benefitting from high accessibility. This result is consistent with the findings of previous studies [53]. Except for a few cases, no significant regional accessibility differences occurred in remote areas. The improvement could be attributed to consistent policies and efforts to reduce regional disparities [69,70]. 

Most studies found that urban or plain areas had higher spatial accessibility to health services than rural or mountainous areas. This spatial discrepancy could be attributed to fundamental differences in geography and economic and social development [57,71,72]. This study also indicated a gradient of community-hospital accessibility, gradually decreasing from the central urban area to the urban edge. This pattern is similar to previous studies in other Chinese provinces and cities. The accessibility to medical resources in economically underdeveloped areas has remained relatively low. In particular, the accessibility to primary health services is still limited, with notable regional differences [73,74,75].

In this study, because of the high spatial accessibility of Niulanshan Town and Nancai Town Community Hospitals, the average accessibility in Shunyi was lifted to a level higher than in Miyun. The Niulanshan Town Community Hospital had an exceptionally high attractiveness, exceeding most counterparts in the study area. The Nancai Town Community Hospital also had high attractiveness. The nearby Nancai Village accommodates many elderly residents who have convenient access to it. Nevertheless, the accessibility to other community hospitals in Shunyi is low. This is because Shunyi is a new urban development area characterized by new industrial establishments and a recent population influx. Our field investigations found that some community hospitals are under construction there. 

The accessibility to community hospitals in Miyun was also low, mainly due to the low population density and economic and social development. Miyun has a mountainous topography, which hinders urban development. Due to the low-density and dispersed settlements, the distances between villages and community hospitals are long, aggravating accessibility to primary medical facilities. In the future, more community hospitals should be allocated in Miyun at strategic gap-filling locations to overcome the unfair distribution induced by inherent geographical barriers. 

Shunyi and Miyun diverged significantly in the attractiveness of their community hospitals but not in spatial accessibility. This is because both districts had similar low accessibility to community hospitals. Although suburban medical facilities are better than those in rural ones, the distance between villagers and community hospitals is longer.Thus, the spatial accessibility of community hospitals was related to their attractiveness and the number of people living within walking distance (Table 4). For example, in Chaoyang, although the Changying Community Hospital is far from the central urban area and had an average attractiveness, its accessibility index reached the highest value in the region. This is mainly because the Ethnic Homeland, Jiandongyuan, Guanzhuang Xili and other nearby communities have more elderly residents. Our finding concurs with previous studies [14,76], which found that the walking distance to community hospitals for the elderly could significantly affect their accessibility. Therefore, planning community hospitals should include the establishment of optimal locations to serve more residents living within walking distance.

### 4.4. Equity between and within Districts

The Gini coefficients indicated a considerable lack of equity within and between districts. The Gini coefficients and Lorenz curves reflected the uneven spatial distribution of community hospitals. Beijing’s rapid urban expansion has extended the spectrum of distribution and access to community medical services in the districts. The undesirable spatial pattern echoed inefficient and inequitable resource allocation [62]. 

The Lorenz curve showed significant differences in community hospital provision within the districts, regardless of central or suburban location. The distribution of community medical resources signifies unfairness to residents, including the elderly. A previous study indicated similar inequity in the central urban area [77]. Our results highlighted the spatial mismatch between community hospital accessibility and the local population demand. The elderly’s lack of fair access in Shunyi was the worst, mainly due to the district’s incipient urbanization and unevenly dispersed urbanized areas. The overall accessibility of Miyun was low, so the intra-district difference for the elderly was small due to equally poor accessibility for most residents.

### 4.5. Suggestions for Improving Community Hospital Services

As the capital of China, Beijing is relatively well-endowed in medical resources. However, the inherent inter- and intra-district disparities in urbanization and population profile have brought disparities in provision quality, quantity and distribution. The supply and location of community hospitals have room for improvement, especially in suburban and rural areas. Our findings can provide references for policymakers to optimize the delivery of community hospital services. Differences in spatial accessibility of community hospitals among districts, as signified by Gini coefficients and Lorenz curves, can pinpoint inequities, especially those affecting the elderly. Resources can be channeled to places with inadequate supply and spatial accessibility gaps. The proposed adjustments can complement and enhance previous planning methods to optimize resource allocation to the elderly. 

The community hospitals in Dongcheng serve 98.23% of the elderly within walking distance, indicating a sufficient, if not exemplary, provision. The capital core area is positioned to strictly control new medical resources’ scale and relieve non-capital functions. The attention can be focused on improving the quality of existing medical facilities, increasing the medical staff in community hospitals, strengthening medical staff skills, developing more specialist clinical departments, and allocating more resources to community health service stations. The key objective is to boost the overall attractiveness of community hospitals to residents, especially the elderly. 

Chaoyang should devote itself to improving the community hospitals located at the outer edge and build new hospitals to fill the service gaps based on the distribution of the elderly and their communities. For example, the following neighborhoods deserve attention: Beiwei Homeland and Zhenyuan Communities on Laiguangying Street, Asian Games new home Zhuxiyuan Community on Datun Street, Shuangqiao No. 6 Well Community in Heizhuanghu District, Shishilidian Oasis Homeland Community, Sunhe Street Kangying Homeland Community, and Yanxiang Dongli Community on Capital Airport Street. These communities and nearby areas have a concentration of elderly people but lack community medical resources. 

Shunyi is a new urban development area. Currently, nearly 40% of its elderly population cannot walk to community hospitals, and overall accessibility is low. It is necessary to construct more community hospitals at strategic locations to shorten their distances to villages, narrow the supply gap, and increase investments in rural community hospitals in suburban areas. Other measures can raise the number of general practitioners and nurses, widen the range of specialist clinical services, and upgrade essential medical equipment. 

Miyun and other ecological conservation areas’ main development goal is to meet the local residents’ health needs in the context of a low-density rural environment. Besides adding more community hospitals to gap areas, some village clinics can be upgraded to hospitals to shorten the distance to villages. The existing community hospitals’ essential medical equipment and staffing can be improved. Measures can include developing telemedicine and other support systems, strengthening the linkage between community and high-grade hospitals, and narrowing the spatial gap in the supply to overcome the unfair distribution of medical resources caused by geographical barriers. 

## 5. Limitations and Future Research

With the progressive aging of the population, the demand for primary medical services for the elderly is anticipated to increase quickly. Many elderly people have chronic ailments and demand regular diagnosis and treatment. Community hospitals can provide residents with basic public health services, diagnosis and treatment of common and chronic illnesses, and have the advantage of proximity to communities. They are more convenient for the elderly to seek medical services than high-grade hospitals. However, few studies have measured the accessibility of community hospitals for the elderly. This paper evaluated four Beijing districts with different intensities of urban development and investigated the accessibility and attractiveness of community hospitals to the elderly group. 

As a leader in China’s health care reform, Beijing’s allocation of medical resources can provide a model for other cities in China and other countries undergoing rapid urbanization. This study could contribute to the literature in two domains. Firstly, we used different distance thresholds and attractiveness indices to build the spatial interaction model to measure the spatial accessibility to community hospitals from the perspective of the elderly group on a small scale. Secondly, the results can inform adjustments and directions in the future planning of community hospitals, improve the spatial allocation of community medical resources, and optimize the fair and equitable provision of primary medical services to the elderly. 

Some limitations of this study should be discussed. Firstly, due to the lack of community-level elderly population data, we had to use the surrogate community household and population raster data to estimate this and then correct and validate the data with the street elderly population and the population of selected communities/villages obtained during our field surveys. The estimated population of some communities could compromise the reliability of the results. Secondly, due to the difficulties in data acquisition, we had to accept a smaller-than-expected set of indicators to compute the attractiveness indices of community hospitals. Future studies can be improved by considering more factors, including the number of doctors and nurses, quantification of clinicians’ professional skills, subdivision of departments, presence of geriatric departments, inventory of key medical equipment, etc. 

Our study chose the Lorenz curve to assess inequalities within and between districts. Other spatial econometric indicators could supplement the findings in subsequent studies. In addition, the accessibility of community hospitals is affected by spatial location, client-facility distance and service capacity, and the subjective perception of community hospitals by the elderly, which can truly reflect holistic and realized accessibility. Therefore, follow-up studies can verify and cross-reference the results of spatial accessibility by a questionnaire survey and other methods. The association between spatial accessibility and residents’ health can provide another fertile research topic. A questionnaire survey can glean the health and medical conditions of residents. By comparing spatial accessibility with perceived accessibility, designs to optimize community hospital accessibility can be further refined. 

## Figures and Tables

**Table 1:** Population size and aging rate (2020) in four Beijing districts chosen as study areas.

District	Total Population (*n*)	Population Aged ≥60 (*n*)	Aging Rate (%)
Beijing	21,890,000	4,299,000	19.6%
Dongcheng	708,829	187,528	26.4%
Chaoyang	3,452,460	708,869	20.5%
Shunyi	1,324,044	218,751	16.5%
Miyun	527,683	122,106	23.1%

Data source: Seventh census of the districts, available at the website https://banshi.beijing.gov.cn/ accessed on 14 October 2022 [46].

**Table 2:** The aged population and communities served by community hospitals within walking distance in four districts.

District	Community Hospitals	Total Aged Population	Served Aged Population		Total Communities	Served Communities	
	(*n*)	(*n*)	(*n*)	%	(*n*)	(*n*)	%
Dongcheng	55	146,977	144,379	98.23	1166	1121	96.14
Chaoyang	212	749,419	672,283	89.70	1818	1572	86.47
Shunyi	104	243,377	140,560	57.75	451	180	39.91
Miyun	39	97,086	65,195	67.15	468	157	33.55
Total	410	1,236,853	1,022,417	82.66	3903	3030	77.63

**Table 3:** Spatial accessibility index of four districts.

Spatial Accessibility	Max (%)	Min (%)	Median (%)	Mean (%)	Q1 ^a^	Q3 ^a^
Dongcheng	4.6418	0.0045	0.0290	0.2438	0.1321	0.7914
Chaoyang	2.0902	<0.01	0.0252	0.0880	0.0104	0.0617
Shunyi	1.2920	<0.01	0.0005	0.0276	<0.01	0.0028
Miyun	0.0914	<0.01	0.0011	0.0054	<0.01	0.0028
Total	4.6418	<0.01	0.0857	0.0857	0.0006	0.0395

^a^ The mean spatial accessibility results were ranked in descending order. Q1 means the first quartile, and Q3 the third quartile.

**Table 4:** Spearman correlation coefficients between accessibility index, attractiveness index and associated factors.

CC	$R_{Ai}$	$R_{Di}$	$R_{Pi}$	$R_{FDi}$	Attractiveness Index	$P/d^{2}$
Accessibility index	0.348 **	0.562 **	0.589 **	0.541 **	0.614 **	0.876 **

** Correlation is significant at the 0.01 level (two-tailed); $R_{Ai}$: relative value of primary health institution area; $R_{Pi}$: relative value of health care personnel number; $R_{Di}$: relative value of department number;$\;R_{FDi}:$ relative value of family doctor team number; $P/d^{2}$: aged population to distance ratio.
